# The Pho23-Rpd3 histone deacetylase complex regulates the yeast metabolic transcription factor Stb5

**DOI:** 10.17912/micropub.biology.000940

**Published:** 2023-08-25

**Authors:** Elizabeth Delorme-Axford, Tabassum Ahmad Tasmi, Daniel J. Klionsky

**Affiliations:** 1 Biological Sciences, Oakland University, Rochester, Michigan, United States; 2 Life Sciences Institute and Department of Molecular, Cellular, and Developmental Biology, University of Michigan–Ann Arbor, Ann Arbor, Michigan, United States

## Abstract

Macroautophagy/autophagy is an essential catabolic process for maintaining homeostasis and cell survival under stressful conditions. We previously characterized the metabolic transcription factor Stb5 as a negative modulator of autophagy through its regulation of genes involved in NADPH production. However, the molecular mechanisms regulating *STB5* expression are not fully characterized. Here, we identify the yeast Pho23-Rpd3 histone deacetylase complex as a transcriptional regulator of *STB5* . Our work provides insight into the mechanisms modulating the metabolic transcription factor Stb5 and expands on the repertoire of genes targeted by the Pho23-Rpd3 complex.

**Figure 1. The Pho23-Rpd3 histone deacetylase complex regulates STB5:**
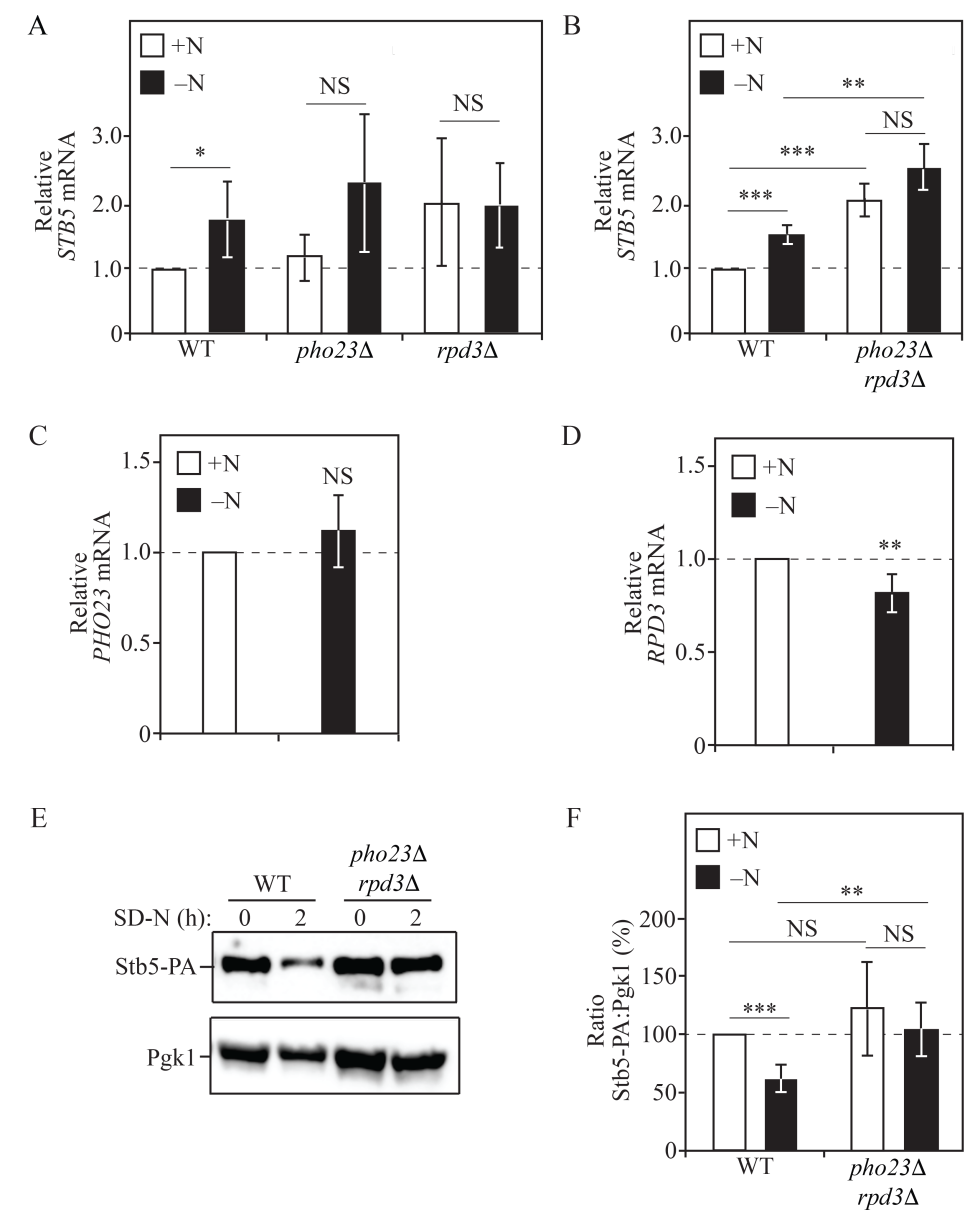
( **A** ) WT (WLY176), *pho23* ∆ (JMY048), and *rpd3* ∆ (JMY093) cells were grown to mid-log phase in YPD (+N) and then nitrogen-starved (–N) for 1 h. Total RNA was extracted, and RT-qPCR was performed. Results are shown relative to the level of *STB5* mRNA expression in WT cells under rich conditions (+N), which was set to 1. *TFC1* was used to quantify relative expression levels (n=4). ( **B** ) WT (WLY176) and *pho23* ∆ *rpd3* ∆ (JMY094) cells were grown to mid-log phase in YPD (+N) and then nitrogen-starved (–N) for 1 h. Total RNA was extracted and analyzed as in ( **A** ). Results are shown relative to the level of *STB5* mRNA expression in WT cells under rich conditions (+N), which was set to 1. *TFC1* was used to quantify relative expression levels (n=4). ( **C** ) WT (SEY6210) cells were grown to mid-log phase in YPD (+N) and then nitrogen-starved (–N) for 1 h. Total RNA was extracted and analyzed as in ( **A** ). Results are shown relative to the level of *PHO23* mRNA expression under rich conditions (+N), which was set to 1. The geometric mean of *TFC1* and *SLD3* was used to quantify relative expression levels (n=3). ( **D** ) WT (SEY6210) cells were grown to mid-log phase in YPD (+N) and then nitrogen-starved (–N) for 1 h. Total RNA was extracted and analyzed as in ( **A** ). Results are shown relative to the level of *RPD3* mRNA expression under rich conditions (+N), which was set to 1. The geometric mean of *TFC1* and *SLD3* was used to quantify relative expression levels (n=4). ( **E** ) WT (EDA216) and *pho23* ∆ *rpd3* ∆ (EDA251) cells endogenously expressing Stb5-PA were grown to mid-log phase in YPD and then starved for nitrogen (–N) for the indicated time points. Protein extracts were analyzed by SDS-PAGE and blotted using an antibody that recognizes PA. Pgk1 is the loading control. The blot shown is representative of 5 independent experiments. ( **F** ) Densitometry of blots represented in ( **E** ). The percentage of Stb5-PA:Pgk1 was quantified (n=5). In ( **A** )–( **D** ) and ( **F** ), results are shown as the mean. Error bars indicate standard deviation (* *p* <0.05; ** *p* <0.01; *** *p* <0.001).

## Description

Autophagy is an essential catabolic process for maintaining homeostasis and cell survival under stress. We previously identified the yeast metabolic transcription factor Stb5 as a negative modulator of autophagy through its regulation of genes involved in reduced nicotinamide adenine dinucleotide phosphate (NADPH) production (Delorme-Axford, Wen et al. 2023) . Stb5 is a C6 zinc cluster DNA-binding protein that has dual functions as either a transcriptional activator or repressor to modulate cellular responses (reviewed in Delorme-Axford, Wen et al. 2023). Most notably, loss of *STB5* decreases cellular NADPH pools (Ouyang, Holland et al. 2018, Delorme-Axford, Wen et al. 2023) . NADPH is a major source of reducing equivalents in the cell for maintaining redox and anabolic reactions (Cao, Wu et al. 2020; and reviewed in Ju, Lin et al. 2020) .

In our previous study, we found that Stb5 protein levels substantially decrease during nitrogen starvation and autophagy induction, consistent with the role of Stb5 as a negative autophagy regulator (Delorme-Axford, Wen et al. 2023) . Nitrogen starvation is a robust stimulus to induce autophagy in yeast (Delorme-Axford, Guimaraes et al. 2015) . Negative autophagy regulators, which maintain autophagy activity at a basal level under nutrient-replete conditions, are typically inactivated following autophagy induction (reviewed in He and Klionsky 2009). Thus, we typically observe expression levels of these negative regulatory factors to decrease under autophagy-promoting (starvation) conditions. In contrast to Stb5 protein levels, we also observed that *STB5* transcript levels increase when cells enter starvation conditions (Delorme-Axford, Wen et al. 2023) . Given the differences that we observed between the protein and mRNA levels during nitrogen starvation and autophagy induction, we sought to identify potential transcriptional regulators of *STB5* .

The Rpd3 large (Rpd3L) complex is one of three Rpd3 complexes that regulate gene expression in yeast (Vidal and Gaber 1991, Carrozza, Florens et al. 2005, Baker, Ueberheide et al. 2013) . Rpd3L is recruited to gene promoters and deacetylates histones H3 and H4 to repress gene transcription (reviewed in Li, Mei et al. 2023). The Rpd3L corepressor complex is comprised of multiple components (Carrozza, Florens et al. 2005) , including several previously identified transcriptional regulators of autophagy such as Ume6 and the Sin3-Rpd3 complex (Bartholomew, Suzuki et al. 2012) , Pho23 and Rpd3 (Jin, He et al. 2014, Cui, Parashar et al. 2019) , and Ash1 (Delorme-Axford, Abernathy et al. 2018) . Ume6 transcriptionally represses *ATG8,* thereby negatively regulating autophagy (Bartholomew, Suzuki et al. 2012) . Transcriptional repression by Ume6 requires the recruitment of the Sin3-Rpd3 complex involving the corepressor Sin3 and the histone deacetylase Rpd3 (Kadosh and Struhl 1997, Bartholomew, Suzuki et al. 2012) . In addition, deletion of any single gene from the complex – *UME6* , *RPD3* , or *SIN3* – derepresses Atg8 expression (Bartholomew, Suzuki et al. 2012) . Interestingly, Stb5 has been reported to interact with Sin3, a component of the Rpd3L complex, in the yeast two-hybrid assay (Kasten and Stillman 1997) .

Pho23 transcriptionally regulates the gene encoding the lipid scramblase Atg9, and thereby modulates the frequency of autophagosome formation during macroautophagy (Jin, He et al. 2014) . Expression of the reticulophagy receptor Atg40 is regulated by Pho23 and Rpd3 (Cui, Parashar et al. 2019) . Ash1 positively regulates the exonuclease Xrn1, and cells lacking *ASH1* show enhanced autophagy activity, supporting its role as a negative modulator of autophagy (Delorme-Axford, Abernathy et al. 2018) . Recent work indicates that Rpd3L-mediated deacetylation is promoted by the Tor complex 1 (TORC1) pathway, which is inactivated under nitrogen starvation conditions to induce autophagy (Li, Mei et al. 2023) . It has also been postulated that Rpd3L may regulate additional unknown proteins involved in autophagy (Li, Mei et al. 2023) .

Following up on our previous work (Delorme-Axford, Wen et al. 2023) , we investigated potential transcriptional regulators of *STB5* . Considering that components of the Rpd3L complex (Bartholomew, Suzuki et al. 2012, Jin, He et al. 2014, Delorme-Axford, Abernathy et al. 2018) and Stb5 (Delorme-Axford, Wen et al. 2023) negatively modulate autophagy, we investigated whether the Rpd3-Pho23 complex regulates *STB5* ; we assessed *STB5* mRNA levels under both growing (+N) and nitrogen starvation (–N) conditions in *pho23* ∆ or *rpd3* ∆ cells with real-time quantitative PCR (RT-qPCR; Fig. 1A ). As expected, we found that *STB5* mRNA levels were significantly enhanced in wild-type (WT) cells at 1 h of nitrogen starvation ( Fig. 1A and B ), consistent with our previous work (Delorme-Axford, Wen et al. 2023) . However, we did not observe a significant difference in *STB5* mRNA levels under either nutrient-rich or nitrogen-starved conditions in cells lacking either *PHO23* or *RPD3* alone ( Fig. 1A ).

Because both Pho23 and Rpd3 are components of the Rpd3L corepressor complex and an impact on *STB5* expression was not clear using a single gene knockout ( Fig. 1A ), we examined *STB5* expression in cells lacking both *PHO23* and *RPD3* ( Fig. 1B ). *STB5* levels were significantly upregulated under both nutrient-rich and nitrogen-starved conditions in cells lacking both *PHO23* and *RPD3* ( Fig. 1B ), supporting the idea that the Rpd3-Pho23 complex transcriptionally modulates *STB5* . In addition, we observed that *PHO23* mRNA levels were not significantly changed, and *RPD3* mRNA levels decreased slightly during nitrogen starvation and autophagy induction ( Fig. 1C and D, respectively ).

To determine how Stb5 protein levels could be affected in *pho23* ∆ *rpd3* ∆ cells, *STB5* was chromosomally tagged with protein A (PA) at its C terminus. Endogenous Stb5 fusion protein levels were assessed in WT and *pho23* ∆ *rpd3* ∆ cells under nutrient-replete and nitrogen-starved conditions by western blot ( Fig. 1E and F ). Consistent with our previous work (Delorme-Axford, Wen et al. 2023) , Stb5-PA protein levels significantly decreased during nitrogen starvation in WT cells ( Fig. 1E and F ). Under nutrient-replete conditions, no significant differences were observed between WT and *pho23* ∆ *rpd3* ∆ cells by western blot ( Fig. 1E and F ). Furthermore, the level of Stb5-PA in *pho23* ∆ *rpd3* ∆ cells did not change significantly between rich and starvation conditions ( Fig. 1E and F ). In contrast, Stb5-PA fusion protein levels were significantly upregulated in *pho23* ∆ *rpd3* ∆ cells compared to WT under starvation conditions ( Fig. 1E and F ), consistent with our qRT-PCR results ( Fig. 1B ).

Here we present data supporting a role for the Pho23-Rpd3 histone deacetylase complex in the transcriptional regulation of *STB5.* Under nutrient-rich conditions, *STB5* mRNA levels are upregulated in cells lacking *PHO23* and *RPD3* . When cells are starved for nitrogen, both *STB5* mRNA and fusion protein levels are higher in *pho23* ∆ *rpd3* ∆ cells. Our previous work showed that the downregulation of Stb5 during nitrogen starvation was dependent on the fundamental autophagy regulator Atg1 and the vacuolar protease Pep4/proteinase A (Delorme-Axford, Wen et al. 2023) . In this study, we provide additional evidence for the transcriptional control of *STB5* – a gene that maintains cellular NADPH levels – by the Pho23-Rpd3 histone deacetylase complex. In addition, this work also expands on the repertoire of Pho23-Rpd3 transcriptional targets previously described by others (Jin, He et al. 2014, Cui, Parashar et al. 2019, Li, Mei et al. 2023) . Whether there are other yet unidentified transcriptional targets that are involved in autophagy that may be regulated by Pho23, Rpd3, or any of the Rpd3-associated complexes remains to be determined.

## Methods

***Yeast strains, media, and cell culture:*** Yeast cells were grown in YPD (1% yeast extract, 2% peptone, and 2% glucose). Autophagy was induced by shifting mid-log phase cells from rich medium to nitrogen starvation medium (SD-N; 0.17% yeast nitrogen base without ammonium sulfate or amino acids and 2% glucose) for the indicated times. Gene deletions and chromosome tagging were performed using standard methods (Longtine, McKenzie et al. 1998, Gueldener, Heinisch et al. 2002) .

***SDS-PAGE and western blot:*** SDS-PAGE and western blots were performed as previously described (Cheong and Klionsky 2008) . Western blots were visualized using an Azure 600 (Azure Biosystems) imaging system. Densitometry for western blots was performed using ImageJ ( https://imagej.nih.gov/ij/ ). A commercial antibody that recognizes the PA tag was purchased from Jackson Immunoresearch (323-005-024). Monoclonal Pgk1 antibody was purchased from Invitrogen (22C5D8).

***RNA and real-time quantitative PCR (RT-qPCR):*** Yeast cells were cultured in YPD to mid-log phase and then shifted to SD-N (1 h) for autophagy induction. Cells (1 OD _600_ unit) were then collected, and the pellets were flash frozen in liquid nitrogen. Total RNA was extracted using an RNA extraction kit (Clontech Nucleo Spin RNA/Takara). Reverse transcription was carried out using the High-Capacity cDNA Reverse Transcription Kit (Applied Biosystems/Thermo Fisher Scientific). For each sample, 1 µg RNA was used for cDNA synthesis. RT-qPCR was performed using the Power SYBR Green PCR Master Mix (Applied Biosystems/Thermo Fisher Scientific) in a Bio-Rad CFX Connect real-time PCR machine. For all RT-qPCR experiments, melt curves were run after the PCR cycles to verify primer specificity. Relative gene expression was calculated using the 2 ^−ΔΔCT^ method (Livak and Schmittgen 2001) and normalized as indicated. RT-qPCR primer sequences are described below.

***Statistical analysis:*** The two-tailed Student’s t test was used to determine statistical significance with GraphPad Prism (GraphPad Software, USA). For all figures, *p* values are as follows: * *p* <0.05; ** *p* <0.01; *** *p* <0.001. A *p* value < 0.05 was considered significant.

## Reagents

**Table:** 

***Saccharomyces cerevisiae* strains used in this study are as follows: **		
**Name**	**Genotype**	**Reference**
EDA216	WLY176, *STB5-PA::KAN*	(Delorme-Axford, Wen et al. 2023)
EDA251	JMY094, *STB5-PA::TRP*	This study
JMY048	WLY176, *pho23* ∆ *::HIS*	(Jin, He et al. 2014)
JMY093	WLY176, *rpd3* ∆ *::HIS*	(Jin, He et al. 2014)
JMY094	WLY176, *pho23* ∆ *::KAN, rpd3* ∆ *::HIS*	(Jin, He et al. 2014)
SEY6210	*MATα his3∆200 leu2-3,112 lys2-801 suc2-∆9 trp1∆901 ura3-52*	(Robinson, Klionsky et al. 1988)
WLY176	SEY6210, *pho13* ∆ *pho8::pho8* ∆ *60*	(Kanki, Wang et al. 2009)
**RT-qPCR primer sequences used in this study are as follows:**		
**Name**	**Sequence (5' – 3')**	**Reference**
*PHO23-F*	CCAACGCTGCCAATACCAAC	This study
*PHO23-R*	TAGTGCTTGGTGAAACGGCT	This study
*RPD3-F*	ACACCTAGGGATGCCGAAGA	This study
*RPD3-R*	CAACATGTAGGTCCCTCGCA	This study
*SLD3-F*	CGCAACTTCAAAGCATCATTGAATCGC	(Bernard, Jin et al. 2015)
*SLD3-R*	GGGGCTTATTAGTGGGAGTAGAGG	(Bernard, Jin et al. 2015)
*STB5-F*	GCGGTTATTACCCTTGAACTGGA	(Delorme-Axford, Wen et al. 2023)
*STB5-R*	TCCGCGCATCTCATAGCAAA	(Delorme-Axford, Wen et al. 2023)
*TFC1-F*	GCTGGCACTCATATCTTATCGTTTCACAATGG	(Teste, Duquenne et al. 2009)
*TFC1-R*	GAACCTGCTGTCAATACCGCCTGGAG	(Teste, Duquenne et al. 2009)
